# High-Salt Diet Accelerates Neuron Loss and Anxiety in *APP/PS1* Mice Through Serpina3n

**DOI:** 10.3390/ijms252111731

**Published:** 2024-10-31

**Authors:** Kaige Ma, Chenglin Zhang, Hanyue Zhang, Chanyuan An, Ge Li, Lixue Cheng, Mai Li, Minghe Ren, Yudan Bai, Zichang Liu, Shengfeng Ji, Xiyue Liu, Jinman Gao, Zhichao Zhang, Xiaolin Wu, Xinlin Chen

**Affiliations:** 1 Department/Institute of Neurobiology, School of Basic Medical Sciences, Xi’an Jiaotong University, Xi’an 710061, China; makaige@xjtu.edu.cn (K.M.); zhangchenglin033@stu.xjtu.edu.cn (C.Z.); zhanghy00@stu.xjtu.edu.cn (H.Z.); 2094213936@stu.xjtu.edu.cn (C.A.); 2206124531@stu.xjtu.edu.cn (G.L.); 2206124543@stu.xjtu.edu.cn (L.C.); 2213621725@stu.xjtu.edu.cn (M.L.); 2225013791@stu.xjtu.edu.cn (M.R.); baiyudan020@gmail.com (Y.B.); limpid@stu.xjtu.edu.cn (Z.L.); jsf850@xjtu.edu.cn (S.J.); xiyueliu@stu.xjtu.edu.cn (X.L.); 2327245856@stu.xjtu.edu.cn (J.G.); zhangzhichao@xjtu.edu.cn (Z.Z.); 2 Key Laboratory of Ministry of Education for Environment and Genes Related to Diseases, Xi’an Jiaotong University, Xi’an 710061, China; 3 Department of Human Anatomy, Histology and Embryology, School of Basic Medical Sciences, Xi’an Jiaotong University, Xi’an 710061, China; 4 Institute of Neuroscience, Translational Medicine Institute, Xi’an Jiaotong University, Xi’an 710061, China

**Keywords:** high-salt diet, Alzheimer’s disease, Serpina3n, neuron death, hippocampus

## Abstract

High salt (HS) consumption is an independent risk factor for neurodegenerative diseases such as dementia, stroke, and cerebral small vessel disease related to cognitive decline. Recently, Alzheimer’s disease-like pathology changes have been reported as consequences of a HS diet in wild-type (wt) mice. However, it has not been revealed how HS diets accelerate the progress of Alzheimer’s disease (AD) in *APP*/*PS1* mice. Here, we fed *APP*/*PS1* mice a HS diet or normal diet (ND) for six months; the effects of the HS/ND on wt mice were also observed. The results of our behavior test reveal that the HS diet exacerbates anxiety, β-amyloid overload, neuron loss, and synapse damage in the hippocampi of *APP*/*PS1* mice; this was not observed in HS-treated wt mice. RNA sequencing shows that nearly all *serpin* family members were increased in the hippocampus of HS-treated *APP*/*PS1* mice. Gene function analysis showed that a HS diet induces neurodegeneration, including axon dysfunction and neuro-ligand-based dysfunction, and regulates serine protein inhibitor activities. The mRNA and protein levels of *Serpina3n* were dramatically increased. Upregulated Serpina3n may be the key for β-amyloid aggregation and neuronal loss in the hippocampus of HS-treated *APP*/*PS1* mice. Serpina3n inhibition attenuated the anxiety and increased the number of neurons in the hippocampal CA1(cornu ammonis) region of *APP*/*PS1* mice. Our study provides novel insights into the mechanisms by which excessive HS diet deteriorates anxiety in AD mice. Therefore, decreasing daily dietary salt consumption constitutes a pivotal public health intervention for mitigating the progression of neuropathology, especially for old patients and those with neurodegenerative disease.

## 1. Introduction

Salt intake above recommended daily intakes is a universal diet habit all around the world [1,2,3]. There is strong and consistent evidence from animal and clinical studies illustrating that high salt (HS) consumption is an independent risk factor for stroke, hypertension, and type 2 diabetes. The report of a joint Food and Agriculture Organization of the United Nations recommends that thes sodium intake of adults should be <85 mmol/day (2 g/d). In the northwest of China, the salt intake of the population is about three times the recommended level. Salt intake also tends to increase with age [3,4].

Recently, it was reported that a HS diet is associated with [5,6,7] Alzheimer’s disease (AD) -like changes, such as cognitive decline [7], inflammation, and increased tau hyperphosphorylation in the brain [8,9,10]. Therefore, HS consumption as a dietary habit influences a number of populations, such as individuals with dementia. AD is the most common form of dementia among older adults [10,11]. Neurons in the hippocampal CA1 (cornu ammonis) region have been implicated in anxiety and memory, associated with AD. β-Amyloid (Aβ) plaques consisting of the extracellular accumulation of abnormally folded Aβ with 40 or 42 amino acids (Aβ40 and Aβ42) are one of the key neuropathological hallmarks of AD [12]. The increased levels of Aβ42 peptides aggregate in soluble oligomers. Prolonged exposure to these peptides induces irreversible damage to synapses and eventually leads to neuron death [10,12]. However, the effects of a HS diet on these neurons as well as behavioral changes in the context of AD have not yet been elucidated.

Serpins are serine protease inhibitors that belong to the protease inhibitor superfamily. They are involved in various physiological and pathological functions, including inflammation, cell migration and differentiation, hormone formation and transport, cell–matrix reconstruction, blood pressure regulation, and intracellular protein hydrolysis. Most serpins are secreted into the extracellular space, except Serpin B. Moreover, serpin A is widely expressed in various tissues. Serpina3 is involved in the progression of AD [13,14]. It is consistently overexpressed in the frontal cortex of patients with early-stage AD-related pathology [15,16]. Furthermore, a high concentration of serpin A3/serpina3n has been found in the cerebrospinal fluid (CSF) and brain of patients with AD [17,18]. However, it is not yet clear whether serpin A3 plays a role in the negative effects of a HS diet in the context of AD.

In this study, we fed *APP*/*PS1* mice with a HS diet or a normal-salt diet for six months. We found that the HS diet increased anxiety and cognitive decline and aggregated the neuron loss in the hippocampal CA1 region in *APP*/*PS1* mice. We also performed RNA sequencing (RNA-seq) to determine the mechanism by which a HS diet causes these aforementioned effects. This analysis revealed that serpina3n plays an important role in mediating neuron loss in the hippocampus of HS-treated *APP*/*PS1* mice. Our findings underscore the importance of controlling salt intake to slow down the progress of neurodegeneration in AD patients.

## 2. Results

### 2.1. Water and Food Intake, Body Weight, and Blood Pressure Changes in APP/PS1 Mice After Six Months of a High-Salt Diet

**Figure 1:**
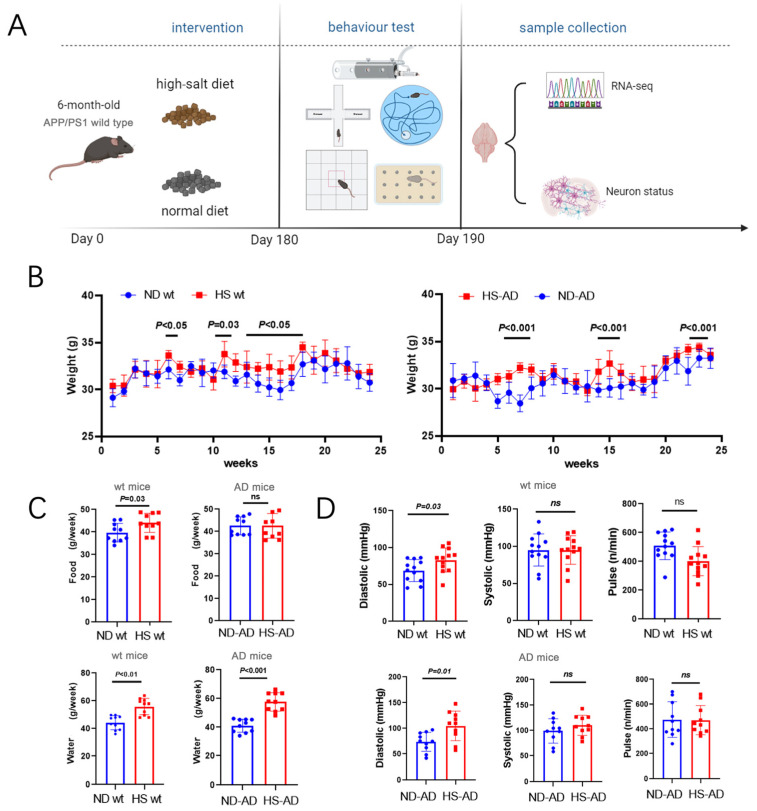
Water intake, food intake, body weight, and blood pressure of AD mice under HS treatment. (**A**) Diagrammatic instructions of the experimental design. *APP*/*PS1* mice (AD mice) or wt mice were fed a HS or ND, and different parameters were measured weekly. (**B**) Time courses of body weight of wt mice and AD mice under HS diet- and ND-treated groups; *n* = 5 in both groups. (**C**) The average water intake of each mouse per week in ND- and HS diet-treated groups. The average food intake of each mouse per week in ND- and HS diet-treated groups; *n* = 10 in each group. (**D**) Pulse, systolic blood pressure, and diastolic blood pressure were measured in conscious, unrestrained mice; *n* = 10 in each group. Blue dots represent the ND treated mice, red blocks represent the HS treated mice.

To investigate the effect of a HS diet on AD mice, *APP*/*PS1* mice were fed a HS diet (8% NaCl) or Normal diet (ND) (0.4% NaCl). The body weight and brain weight, as well as the water and food intake, of the mice were monitored. Additionally, a series of animal behavior tests and subsequent sample analyses were performed (Figure 1A). The results implied that the HS significantly increased body weight in both wt and *APP*/*PS1* mice. Body weight was significantly increased in the sixth, eleventh, and thirteenth to seventeenth weeks in wt mice (*P* < 0.05), while in AD mice, it was significantly higher in the HS group than the ND group in the 6th to 8th, 15th, and 24th weeks (*P* < 0.001, Figure 1B). Compared with the ND group of wt mice, the HS group showed significantly increased water and food intake (*P* < 0.05, Figure 1C). The water intake was markedly lower in the HD-AD group than the ND-AD group (*P* < 0.001), while the difference in food intake between HD-AD and ND-AD was not significant (*P* > 0.05, Figure 1C). Meanwhile, the systolic pressure was significantly higher in the HS group compared with the ND group between the HS and ND groups in wt and *APP*/*PS1* mice (*P* < 0.05); the pulse and systolic blood pressure did not differ between these two groups in the wt or *APP*/*PS1* mice (*P* > 0.05, Figure 1D).

### 2.2. A High-Salt Diet Exacerbates Cognitive Decline and Anxiety in APP/PS1 Mice

**Figure 2:**
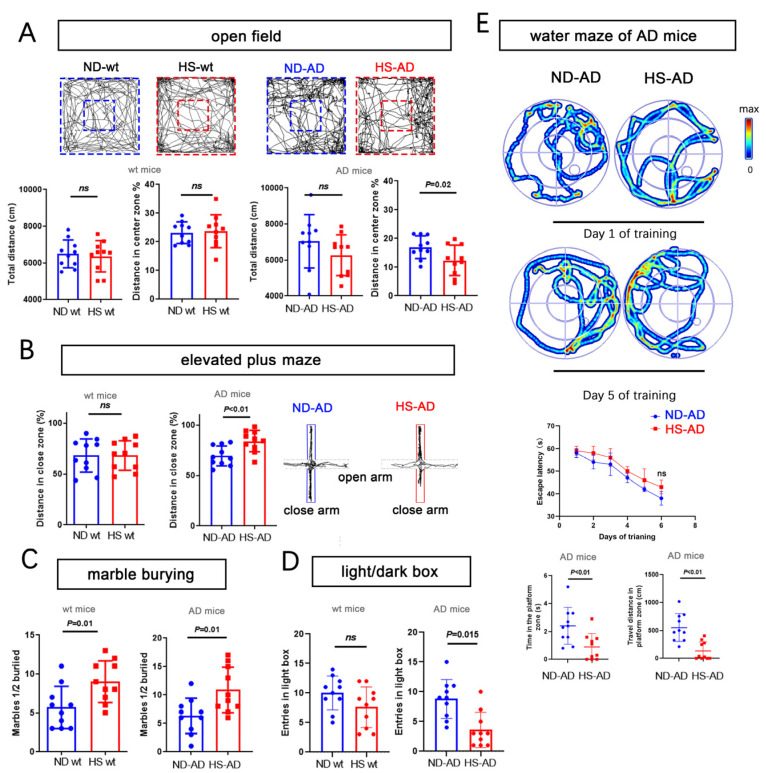
HS diet accelerates anxiety and cognitive decline in AD mice. (**A**) Bar graphs show the total distance traveled, percentage of distance, and time in center zone in the open-field test. Wt mice and AD mice (*APP*/*PS1* mice). *n* = 10 in each group. (**B**) The graph for the elevated-plus maze test shows the mouse movement trajectory in the HS-treated and ND-treated groups. The bar graph shows the traveled distance in the close and open zones in the HS and ND groups, both in wt mice and AD mice. *n* = 10 in each group. (**C**) The bar graph for the marble-burying test shows the marbles buried in the HS and ND groups; *n* = 10 in each group. (**D**) The bar graph for the light/dark box shows the entrance number in the light box in the HS and ND groups; *n* = 10 in each group. (**E**) For the water maze test, the trajectories of mice in the ND-AD and HS-AD groups on day 1 and day 5 of training are shown; *n* = 10 in each group. Escape latency, time spent in the platform zone (the zone in which the platform was placed from day 1 to day 5), and the traveled distance in the platform zone were calculated; *n* = 10 in each group. Blue dots represent the ND treated mice, red blocks represent the HS treated mice.

We next used the open-field test, water maze, elevated-plus maze, marble-burying test, and a light/dark box to assess anxiety-like behavior. For the open-field test, the total distance traveled was similar between the HS and ND groups (*P* > 0.05), while the distance in the enter zone in the ND-AD group was remarkably higher than in the HS-AD group (*P* = 0.02, Figure 2A). The HS-AD group traveled less in the close zone than the ND-AD groups (*P* < 0.01), and there was no significant difference between the HS wt and ND wt groups (*P* > 0.05, Figure 2B). The HS-AD group buried more marbles (*P* = 0.01, Figure 2C) and spent less time in the light box than the ND-AD group (*P* = 0.015, Figure 2D). We also used the water maze test to assess the cognitive status of the mice. Compared with the ND-AD group, the HS-AD group spent less time in the platform zone and traveled a markedly shorter distance in the platform zone (*P* < 0.01, Figure 2E). Taken together, these results indicate that a HS diet exacerbates anxiety in *APP*/*PS1* mice.

### 2.3. A High-Salt Diet Induces Neurodegeneration in the Hippocampal CA1

**Figure 3:**
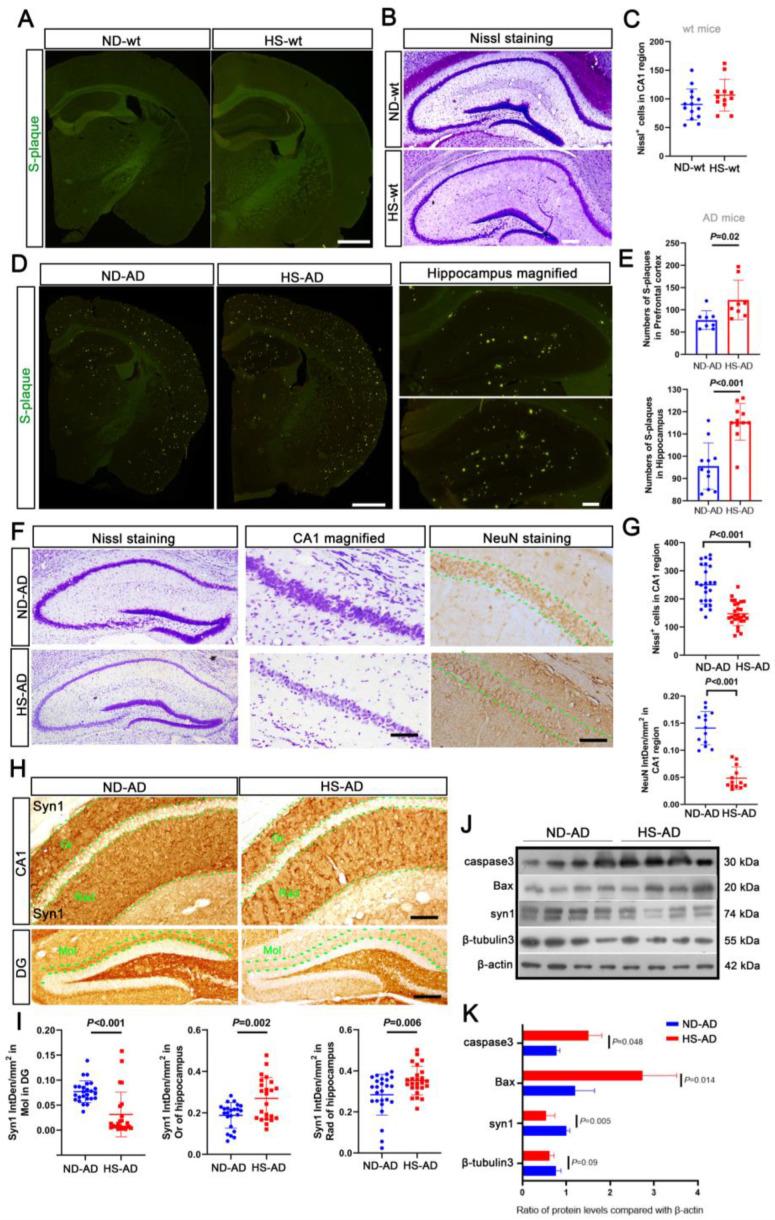
HS diet induces neuron reduction in AD mice. (**A**) Thioflavin T staining was used to detect S-plaque in mice brains. No S-plaque was observed in the HS/ND-treated wt mice; *n* = 5 in each group. Scale bar = 50 μm. (**B**,**C**) Nissl staining was performed to detect the neuron number of HS/ND-treated wt mice. Neuron numbers were counted in the CA1 region of the hippocampus in each group. Scale bar = 50 μm. (**D**,**E**) Thioflavin T staining was used to detect S-plaque in the brains of HS/ND-treated AD mice. The number of S-plaques in parietal cortex, hippocampus, and prefrontal cortex was counted; *n* = 5 in each group. Scale bar = 50 μm. (**F**) Representative Nissl staining of hippocampus in each group. CA1 regions are magnified on the right. The immunohistochemistry for NeuN in the hippocampus of each group is also shown. Between the two green dotted lines is the neurons in the CA1 region. Scale bar = 50 μm. (**G**) The Nissl body-positive neuron numbers were counted from the CA1 region of brain slides from five mice in each group; *n* = 5 in each group. The scatter bar chart shows the integrated density of NeuN-positive particles in each brain slide from five mice, which was measured using Image J software; *n* = 5 in each group. (**H**) Immunohistochemistry for syn1 staining in the hippocampus of each group. Between the two green dotted lines is the neurons in the Or, Rad, and Mol regions; scale bar = 20 μm. (**I**) The scatter bar chart shows the integrated density of Syn1-positive particles from Or, Rad, and Mol, respectively. Each brain slide from five mice was measured using Image J software; *n* = 5 in each group. (**J**,**K**) Western blot analysis for caspase 3, Bax, Syn1, and β-tubulin 3; β-actin was used as an internal control. The data are the mean ± SE; *n* = 4 biologically independent animals. Blue represent the ND treated mice, red represent the HS treated mice.

S-plaque was not observed in the wt mice (Figure 3A). Nissl staining revealed that the hippocampus was densely packed with neurons, with abundant Nissl bodies in the cytoplasm (Figure 3B,C). The HS treatment showed greater Aβ aggregation in the hippocampus and prefrontal cortex of AD mice (*P* < 0.05, Figure 3D,E). Furthermore, in the AD mice, the neurons in the hippocampus were loosely arranged, with some wrinkled cells and fewer Nissl bodies in the HS group (*P* < 0.001), and there were fewer NeuN-positive cells in the CA1 region of the hippocampus in the HS group compared with the ND group (*P* < 0.001, Figure 3F,G). Consistently, there was a decrease in the synapsin I (Syn1)-positive particles in the Or, Rad, and Mol layers of the HS group compared with the ND group (*P* < 0.05, Figure 3H,I). Western blotting revealed that compared with the ND group, there was increased caspase-3 and Bax expression and decreased Syn1 and β-tubulin3 expression in the hippocampus of the HS group (*P* < 0.05, Figure 3J,K). These results indicate that a HS diet induces neuronal degeneration, which may exacerbate anxiety in *APP*/*PS1* mice.

### 2.4. A High-Salt Diet Increases Serpina3n Expression in the APP/PS1 Mouse Brain

**Figure 4:**
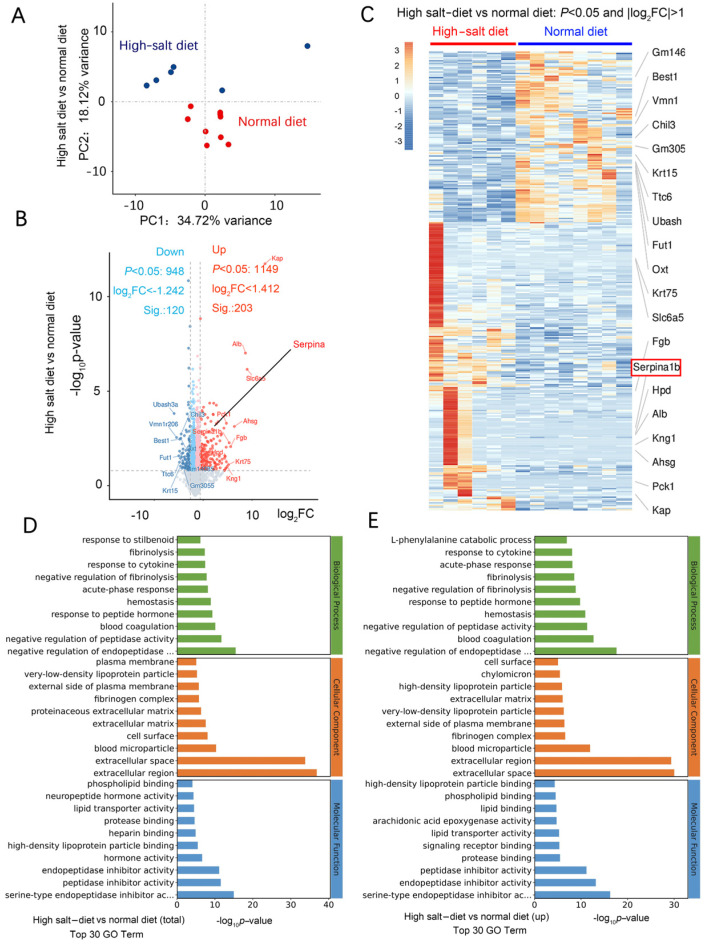
HS diet regulates neuron function-related mRNA expression. (**A**) PCA analysis of data from RNA-seq; samples were collected from the hippocampus of mouse brains in each group. *n* = 6 in HS group; *n* = 7 in ND group. (**B**) Volcano plot of the upregulated (in red) and downregulated (in blue) mRNA. (**C**) Heat map comparing the mRNA expression in the hippocampus from HS and ND mice. *n* = 6 in HS group; *n* = 8 in ND group; |Log_2_FC|>1. (**D**) Results of GO analysis for the total significantly changed mRNA. (**E**) Results of GO analysis for the significantly increased mRNA in the HS-treated group compared to the ND mice.

We used RNA-seq to investigate the mechanism of HS-induced neurodegeneration. A principal component analysis (PCA) showed that the HS diet was responsible for most of the variance (Figure 4A). In the wt mice fed a HS diet, 197 genes, including *Ttr*, *Kcne2*, *Slc4a5*, *Ms4a15*, *S100a5*, *Eomes*, *Mpo*, *Mmp13*, *Hemgn*, and *Elane*, were upregulated compared with the wt mice fed a normal-salt diet. Moreover, there were 38 downregulated genes, including *Pigr*, *Ubash3a*, *Agr2*, *Bcl2a1d*, *B4galnt2*, *Trim40*, *Saa1*, and *Oit*. Most of these are genes expressed in the extracellular region and are related to calcium ion binding. In the HS group, 203 genes were significantly upregulated (Log_2_ fold change > 1.412), including *Kap*, *Alb*, *Slc6a5*, *Ahsg*, *Pck1*, Fgb, *Krt75*, *Kng1*, *serpina1*, *serpina3*, *serpina6*, *serpinb5*, *serpinc1*, *serpine1*, and *serpinf2* (Figure 4B). There were 120 significantly downregulated genes (log_2_ fold change < −1.242), including *Chil3*, *Ubash3a*, *Vmn1r206*, *Best1*, *Fut1*, *Ttc6*, *Krt15*, and *Gm3055* (Figure 4B).

**Figure 5:**
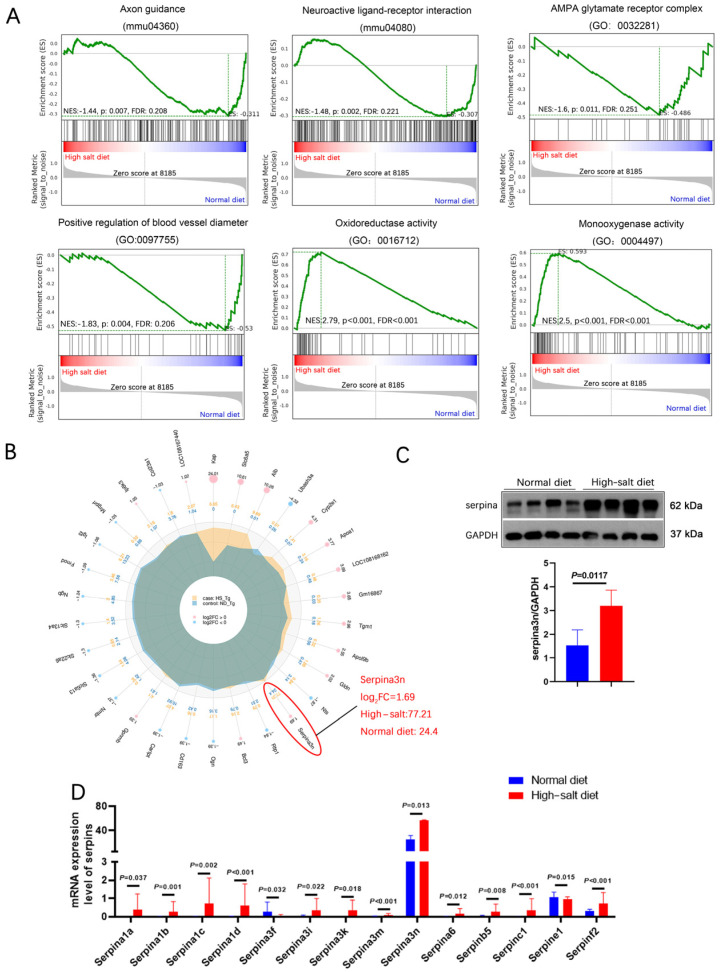
HS diet increased the *serpina3n* expression in the hippocampus of *APP*/*PS1* mice. (**A**) GSEA showed that the HS diet intervention changed the status of axon guidance, neuroactive ligand–receptor interaction, oxidoreductase activity, and blood vessel diameter. (**B**) A radar map of differential gene expression levels. *Serpina3n* is most significantly changed, and its mRNA expression is the highest among the *serpin* family. (**C**) Protein level of *serpina3n* was assessed using Western blotting; GAPDH was used as a control. *n* = 4 biologically independent animals. (**D**) mRNA levels of the *serpin* family in HS- and ND-treated *APP*/*PS1* mice were compared; *serpina3n* is the most remarkably changed mRNA.

Kyoto Encyclopedia of Genes and Genomes (KEGG) pathway enrichment analysis revealed that neuroactive ligand–receptor interaction is the most significant functional change. Gene Ontology (GO) analysis revealed that most of these differentially expressed genes are negative regulators for endopeptidase and function in the extracellular region. Moreover, most of them exhibit peptidase inhibitor activity and serine-type endopeptidase inhibitor activity (Figure 4D,E). Gene set enrichment analysis (GSEA) showed that the HS diet intervention exacerbated the impairment of axon guidance, neuroactive ligand–receptor interaction, oxidoreductase activity, and blood vessel diameter (Figure 5A). These functions are related to blood pressure, neuronal death, and oxidative stress.

We selected the top-30 differentially expressed genes in the HS group and prepared a radar map. It showed that *serpina3n* markedly increased in the HS group (Log_2_FC = 1.69), with a mean expression level of 77.21 in the HS group and 24.4 in the ND group (Figure 5B). In addition, all *serpin* family members (6% of increased genes) except *serpina3f* showed dramatically elevated expression (Figure 5D), which clearly demonstrates that the serpin family may play an important role in HS-induced neurodegeneration in *APP*/*PS1* mice. Furthermore, Western blotting revealed significantly elevated *serpina3n* protein expression in the HS group hippocampus compared with the ND group hippocampus (*P* = 0.012, Figure 5C). Our result indicates that the neurodegeneration induced by a HS diet may be due to the massive upregulation of *serpinA3n* in the hippocampus.

### 2.5. The Serpina3n Inhibitor ARN-1468 Improves the Cognitive Decline and Anxiety Exacerbated by the High-Salt Diet

**Figure 6:**
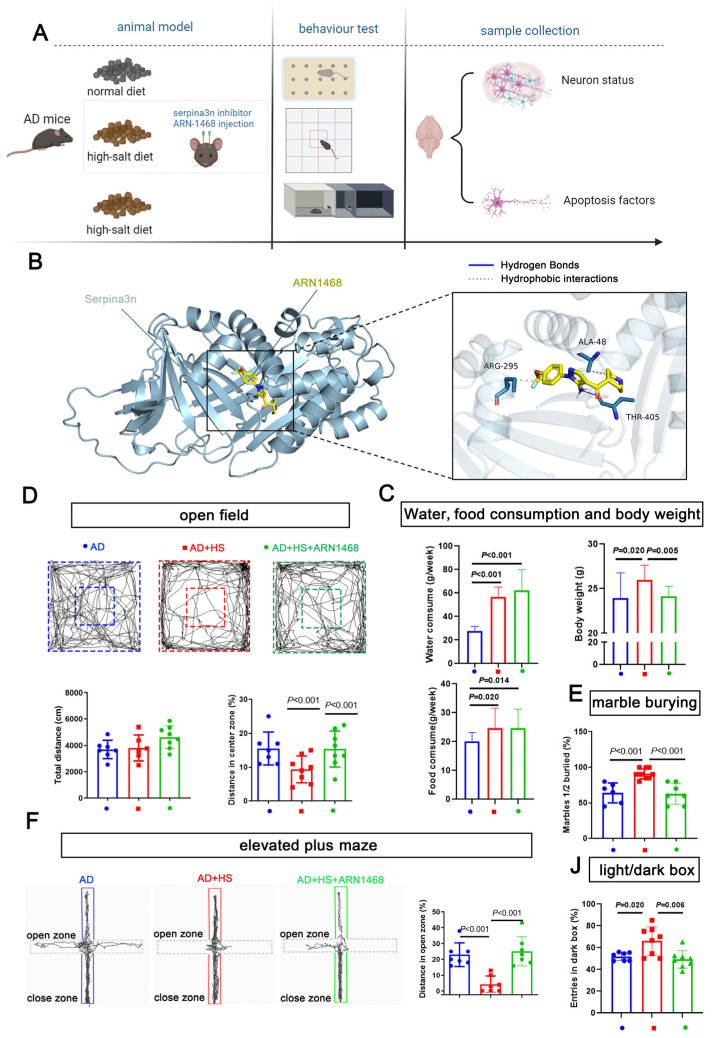
Serpina3n inhibitor alleviates the abnormal anxiety in HS-treated AD mice. (**A**) Diagram illustrating the experimental design for studying the role of serpina3n in the abnormal anxiety observed in HS-treated AD mice. (**B**) Docking simulation revealed that the binding energy of the compound ARN1468 and the protein serpina3n is −5.4 kcal/mol. The specific binding information is shown in (**B**). ARN1468 binds the 405A of serpina3n; they formed a hydrogen bond. (**C**) The average food, water intake, and weight of each mouse per week (in the first 2 months) in the ND- and HS diet-treated groups; *n* = 5 in the ND group and *n* = 5 in the HS group. The bar graph shows the total distance traveled in the open-field test. (**D**) The bar graph shows the percentage of distance traveled in the open zone in the elevated maze test. *n* = 7 in each group. (**E**) Bar graph compares the marbles buried in each group; *n* = 6 in the AD and AD+HS+ARN1468 groups and *n* = 8 in the AD+HS group. (**F**) The graph for the elevated-plus maze test shows the trajectory of mouse movement in the HS-treated group and the ND-treated groups. The bar graph shows the distance traveled in the close and open zones in the three groups: AD (*n* = 6) and AD+HS+ARN1468 (*n* = 6); *n* = 8 in AD+HS (*n* = 8) group. (**J**) The bar graph for the light/dark box shows the number of entries into the light box in the three groups: AD (*n* = 6) and AD+HS+ARN1468 (*n* = 6); *n* = 8 in the AD+HS (*n* = 8) group. Red represents the mice in HS+AD groups, blue represents the mice in AD groups, green represents the mice in HS+AD+ARN1468 groups.

We further evaluated the role of serpina3n in the HS group by stereotactically injecting the serpin inhibitor ARN1468 into the hippocampus of *APP*/*PS1* mice for 14 days after 4 months of being fed a HS diet. The water and food consumption were measured from the day when the ARN1468 injection was started and lasted for 2 months. The body weights of the mice from each group were measured on the day the mice were sacrificed (6 months). This was 2 months after ARN1468 was injected (Figure 6A). ARN-1468 has been shown to inhibit serpina3n [19]. Our docking simulation calculated a binding energy of −5.4 kcal/mol for ARN1468 and serpina3n. As shown in Figure 6B, ARN1468 forms a hydrogen bond with residue 405A of serpina3n. After 4 months of the HS diet, the AD+HS mice were injected with ARN-1468, and the mice in the other two groups were injected with saline for 14 days. Two months after the injection, the AD+HS group consumed more water and food compared with the AD group (*P* < 0.05, Figure 6C), and the body weight increased significantly. However, the AD+HS+ARN1468 group showed a lower body weight compared with the HS+AD group (*P* < 0.05, Figure 6C). Based on the open-field test, all groups traveled the same total distance (*P* > 0.05, Figure 6D). However, the AD+HS+ARN1468 group traveled a greater distance in the center zone compared with the HS+AD group (*P* < 0.01, Figure 6D). In the marble-burying test, the AD+HS+ARN1468 group buried fewer marbles than the AD+HS group (*P* < 0.001, Figure 6E). In the elevated-plus maze test, the AD+HS+ARN1468 group spent more time in the open zone compared with the AD+HS group (*P* < 0.001, Figure 6F). Finally, for the light/dark box, the AD+HS+ARN1468 group spent more time in the light box. Based on these results, ARN1468 attenuated anxiety in HS diet-treated *APP*/*PS1* mice.

### 2.6. The Serpina3n Inhibitor ARN-1468 Alleviates CA1 Neurodegeneration

**Figure 7:**
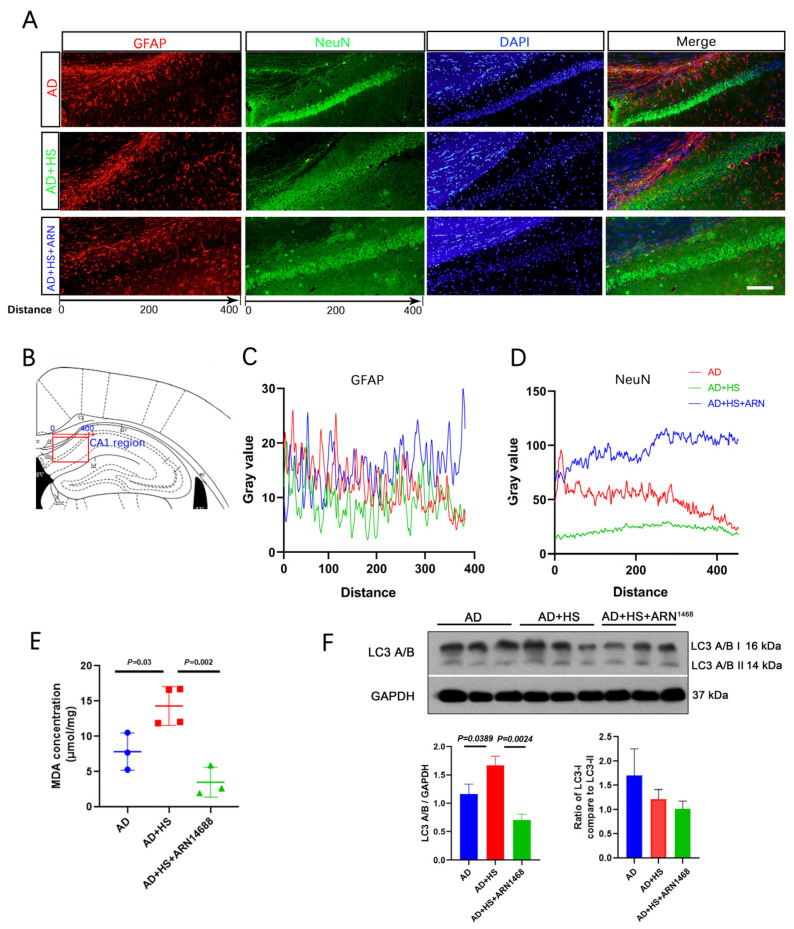
Serpina3n inhibitor increases neuron numbers and reduces cell apoptosis in HS-treated AD mice. (**A**) Representative immunofluorescence images of dual NeuN (green) or GFAP (red) and DAPI (blue) from CA1 of hippocampus; scale bar = 50 μm. (**B**) Graphical depicting the x-axis of the gray value measurement in the three groups. (**C**) Quantitative analysis of NeuN levels among the three groups. (**D**) Quantitative analysis of GFAP levels among the three groups. (**E**) Representative Western blot images of LC3A/B and GAPDH in the hippocampus. (**F**) Quantification of the relative amount of LC3A/B compared to GAPDH and LC3A/B I compared to LC3A/B II in the Western blotting experiment; *n* = 3 biologically independent animals.

We found more NeuN-positive cells in the AD+HS+ARN1468 group compared with the AD and AD+HS groups (Figure 7A,B). The number of GFAP-positive particles did not differ between the three groups (Figure 7C). The level of malondialdehyde (MDA) can reflect the degree of lipid peroxidation in cells and indirectly indicate the extent of cellular damage. We found a lower MDA level in the hippocampus of the AD+HS+ARN1468 group compared with the AD+HS group (*P* = 0.002, Figure 7D). Finally, Western blotting revealed reduced caspase and LC3A/B expression in the AD+HS+ARN1468 group (*P* < 0.05, Figure 7E,F).

## 3. Discussion

Unfavorably HS diets remain prevalent around the world. HS intake can negatively affect both cardiovascular health and brain function [1,20,21]. However, there has been limited research on the long-term damage caused by a HS diet in the context of AD. Thus, we subjected *APP*/*PS1* mice to a HS diet for 6 months to determine whether this treatment accelerated cognitive decline and anxiety.

A previous study showed that, after 16 weeks of a HS diet, salt-sensitive rats showed decreased body weight, accompanied by increased food and water intake and blood pressure [22]. In the present study, the HS group consumed more water and food during the first two months of the HS treatment, but there was minimal change to their body weight. Moreover, the HS group did not show an increase in pulse or systolic blood pressure. These results are consistent with previous studies using the same mouse model [23,24]. We conclude that rodents can exhibit different physiological and behavioral effects even when submitted to the same dietary conditions.

We found that feeding *APP*/*PS1* mice with a HS diet for 6 months elevated anxiety-related behavior and impaired cognitive function. Based on a previous study, a HS diet can alter anxiety and cognitive decline in rats in an age-dependent manner. Specifically, 2- and 20-month-old male rats fed a HS diet (8% NaCl) displayed anxiety-like behavior. It is widely accepted that 5-HT is a key for anxiety. Selective serotonin reuptake inhibitors (SSRIs) could improve behavioral performance [25,26]. Studies in AD mouse models, such as *APP*/*PS1* or Aβ-injected mice, reported that SSRIs restored cognition and cell density in the hippocampus [27]. Treatments with SSRIs or 5-HT receptor agonists reduced Aβ deposition in *APP*/*PS1* mice [28,29]. However, the role of the 5-HT system in the HS-induced neuron loss in the CA1 region of the hippocampus has not yet been revealed. This may be a new direction for our future research. Moreover, short-term memory deficiency was reported in older rats fed a HS diet [30]. In another study, HS intake correlated positively with cognitive decline and anxiety; 12 weeks of feeding a HS diet (7.0% NaCl) significantly impaired spatial memory compared with a normal-salt diet (0.4% NaCl) [23]. When excessive salt is consumed, there is increased circulating interleukin (IL)-17 in the bloodstream; this cytokine prevents vascular endothelial cells from producing nitric oxide. The reduction in nitric oxide levels restricts blood flow to the brain, potentially triggering cognitive impairment and anxiety-like behavior [31]. The dorsal CA1 subregion of the hippocampus is enriched in place cells, and ventral CA1 is enriched in anxiety cells. These anxiety cells were enriched in the vCA1 population projecting to the lateral hypothalamic area but not to the basal amygdala. Therefore, we suggest that the older adult population is more susceptible to the negative effects of high salt intake, including cognitive decline and trouble with regulating emotions. These deficits may be caused by local inflammation in the CA1 region of the hippocampus.

Wt mice fed a HS diet exhibit AD-like cognitive decline as well as tau hyperphosphorylation [21] and neuroinflammation [3]. Notably, the previous studies evaluating the effects of a HS diet lasted less than four months and mostly involved wt mice [31]. Therefore, we focused on a mouse model of AD. We found that a HS diet led to Aβ overload and neuronal loss in the hippocampal CA1 region. In C57BL/6 mice, a HS diet reduced syn1 and postsynaptic density 95 (PSD95) expression, resulting in significant synaptic damage [32]. Chen et al. demonstrated that a HS diet exacerbated cognitive deficits and neurovascular abnormalities in *APP*/*PS1* mice, including damage to the blood–brain barrier, increased permeability of cerebral vessels, and changes in the structure of cerebral vessels [3]. Based on these changes, we aimed to determine the mechanisms by which a HS diet damages the hippocampus of *APP*/*PS1* mice.

The rapid development of RNA-seq has greatly improved our understanding of the molecular pathogenesis of diseases. Kinesin-driven organelle transport is crucial for neuronal development and maintenance: it facilitates organelle transport parameters in axons and dendrites. Kinesin-associated protein (Kap) is a neuronal organelle adaptor to which kinesins bind [33]. Serum albumin (Alb) regulates blood plasma colloid osmotic pressure and acts as a carrier protein for a wide range of endogenous molecules, such as hormones, fatty acids, and metabolites, as well as exogenous drugs. *Slc6a5* encodes the presynaptic glycine transporter 2 (GlyT2); *SLC6A5* mutations result in decreased glycine uptake or both, affecting predicted glycine and Na+ binding sites [34]. *Ahsg* encodes the alpha 2-HS glycoprotein, which is highly related to endocytosis and brain development. *Pck1* regulates gluconeogenesis, and the expression of this gene can be changed by diet. These aforementioned genes showed significantly altered expression in the HS group. These changes illustrate that a HS diet affects metabolic and neural functions in *APP*/*PS1* mice. Of note, these genes mostly encode extracellular proteins.

The GSEA results showed that a HS diet changed the status of axon guidance, neuroactive ligand–receptor interaction, oxidoreductase activity, and blood vessel diameter. These functions are in line with our results regarding the effect of a HS diet on blood pressure, neuronal death, oxidative stress, and cognitive and emotion-related changes in *APP*/*PS1* mice. GO analysis revealed significant changes in the negative regulation of growth and calcium ion binding in wt mice fed a HS diet. However, in the HS group, the changes were related to negative regulation of endopeptidase and serine-type endopeptidase inhibitor activity, most of which function in the extracellular space. Serine inhibitors such as serpin family members are the most broadly distributed superfamily of proteases inhibitors. Serpin A3 has been associated with AD and prion diseases. It was reported that ApoE4 can not only carry out Aβ oligomer transportation but also interacts with Aβ and increases the synthesis of cytokines. A high level of serpina3n is positively related to inflammation and the formation of β-amyloid plaques. There exists a potential that the ApoE4 may contribute to this process [16,17,18,33,34,35]. In another study, the authors found that serpin A3 and serpin B1 are upregulated in patients with AD [36]. Interestingly, we found that serpina3n transcript and protein were upregulated in *APP*/*PS1* mice. Serpina3n, also known as α1-antitrypsin, has various biological functions, including anti-inflammation, anti-oxidation, and anti-apoptosis. In recent years, researchers have focused on the relationship between serpina3n and neurological diseases [37].

We found that the HS group showed increased expression of several serpin genes, including *serpina1a-d*, *serpina3i/k/m/n*, *serpina6*, *serpinb5*, *serpinc1*, *serpine1*, and *serpinf2*, but not *serpina3f*, which was highly decreased. Among these, our correlation network analysis revealed that *serpina3n* is at the center of these altered genes. Serpina3n protein expression was also increased in the HS group. These findings are consistent with a previous study that investigated patients with AD and animal models [36]. Schipanski et al. found that serpina3n is decreased in the CSF and hippocampus of patients with AD, and this decrease was associated with the severity of the disease [38]. In another study, the serpina3n plasma level was associated with Aβ deposition in the brain and cognitive decline among older adults [39]. The Aβ accumulation was further corroborated by serpina3n-dependent prion accumulation. We found a significant increase in serpina3n in the HS group, but not in wt mice fed a HS diet. Serpina3n polymorphisms can prolong the oligomeric state of Aβ, stabilizing its oligomeric form and preventing its transition to higher or lower aggregates [40]. ARN1468 suppresses cellular prion protein (PrPC) expression by inhibiting serpina3n [19]. Our molecular docking experiment showed that ARN1468 binds to serpina3n at residues 405A, 295A, and 48A (with a binding energy of -5.4 kcal/mol). The autophagy marker known as light chain 3 (LC3) was recognized as a component of microtubule-associated proteins 1A and 1B, playing a pivotal role in autophagy [19]. During the autophagy process, LC3-I undergoes a transformation into LC3-II through a lipidation reaction facilitated by a ubiquitin-like system that involves Atg7 and Atg3 enzymes. This reaction enables LC3 to attach to autophagic vesicles [41,42,43]. The detection of LC3 within autophagosomes and the shift of LC3 to its lower migrating form LC3-II serve as indicators of autophagy activity [44]. We found that ARN-1468 treatment reduced neuronal damage, decreased LC3A/B and apoptosis in the hippocampus, and reduced anxiety-like behavior in AD mice fed a HS diet.

Collectively, we demonstrated that a HS diet could induce neuronal loss, suppress the expression of synaptic proteins, lead to Aβ plaque aggregation in the hippocampus, and cause anxiety-like behavior in *APP*/*PS1* mice. Anxiety-like behavior, neuronal loss in the hippocampus, and apoptosis were attenuated by inhibiting serpina3n. This suggests that HS consumption induces neuronal loss and anxiety-like behavior through the upregulation of serpina3n. Our study draws attention to the importance of reducing daily salt intake, especially for patients with Alzheimer’s disease.

## 4. Materials and Methods

### 4.1. Animals

All experimental animal procedures were conducted in accordance with guidelines established by the Animal Committee of Xi’an Jiaotong University Health Science Center (NO.2022-1109, 7 March, 2022). All possible efforts were used to minimize the number of animals used and their suffering. 6-month-old male *APPswe*/*PS1Δe9* (*APP*/*PS1*) mice were used in this study. Male *APP*/*PS1* mice on the C57Bl/6J background were bred with wt C57Bl/6J females (obtained from Wei-Na Yang, Xi’an Jiaotong University, China) at the Xi’an Jiaotong University Animal Center. The offspring were genotyped with the following primers to determine whether they carried the *APPswe*/*PS1Δe9* transgene: forward 5′-GAATTCCGACATGACTCAGG-3′; reverse 5′-GTTCTGCTGCATCTTGGACA-3′. Heterozygous males (i.e., carried one copy of the *APPswe*/*PS1Δe9* transgenic construct) were used. The mice were maintained at 20–25 ± 2 °C and relative humidity (50 ± 15%) with a 12 h light/12 h darkness cycle. All mice were allowed access to food and water ad libitum. At 6 months of age, the mice were divided into two groups. The HS group was fed a HS diet (8% NaCl), while the ND group was fed a ND (0.4% NaCl).

### 4.2. Histochemistry

The mice were anesthetized using sevoflurane. Then they were transcardially perfused with cold 0.01M phosphate-buffered saline (PBS). The brains were removed, fixed in 4% paraformaldehyde, and then transferred to 30% sucrose for 48 h. Tissue sections (14 µm) were cut using a cryostat (CM1950, Leica Microsystems, Wetszlar, Germany).

### 4.3. S-Plaque Staining

Tissue sections were incubated with 0.5% thioflavin-S (dissolved in 50% ethanol) for 30 min. Then the sections were differentiated twice in 80% ethanol (for 10 s each) and then washed with 0.01M PBS.

### 4.4. Nissl Staining

Tissue sections were incubated in toluidine blue stain at 50–60 °C for 25–50 min. Then the sections were rinsed slightly with distilled water and 70% ethanol. The sections were differentiated in 95% ethanol and then washed with distilled water.

### 4.5. NeuN and Synaptophysin (Syn1) Immunohistochemistry

The tissue sections were incubated in hydrogen peroxide and permeabilized using 0.3% Triton-100 (in 0.01 M PBS). After permeabilization, the sections were incubated with 5% normal serum for 2 h to prevent non-specific protein binding. The sections were incubated with rabbit anti-NeuN (1:500, 14H6L24, Invitrogen, CA, USA) or mouse anti-syn1 (1:200, 7H10G6, Novus, CA, USA) overnight at 4 °C. After this incubation, the sections were incubated with biotinylated goat anti-mouse/rabbit IgG working solution for 1 h at room temperature and then washed three times with 0.01M PBS. The sections were incubated with streptavidin working solution for 1h and then freshly prepared diaminobenzidine (DAB) solution to visualize NeuN and Syn1 staining. The percentage area stained in each image was quantified using a multi-threshold plug-in within Image J (National Institutes of Health, Bethesda, MD, USA).

### 4.6. NeuN and Glial Fibrillary Acidic Protein (GFAP) Double Staining

The tissue sections were permeabilized using 0.3% Triton-100 (in 0.01 M PBS). Non-specific staining was blocked with 5% normal goat or donkey serum, depending on the species in which the secondary antibody was raised. The sections were incubated with goat anti-rat GFAP (1:1000, 2.2B10, Invitrogen) and goat anti-rabbit NeuN (1:500, 14H6L24, Invitrogen) for 18 h at 4 °C. After washing, the sections were incubated with goat anti-mouse IgG conjugated to Alexa Fluor 488 (1:800, Invitrogen) and goat anti-mouse IgG conjugated to Alexa Fluor 594 (1:800, Invitrogen) for 2 h at room temperature. The sections were viewed with a fluorescent microscope (BX57, Olympus Corporation, Tokyo, Japan). The number of cells with a positive fluorescence signal was counted with Image J software (version java8).

### 4.7. Behavioral Tests

All the tests were conducted during the dark cycle, beginning 30 min after the lights were turned off and after acclimation for at least 2 h to the testing room, which was dimly lit by a red lamp with a luminosity of <20 lux. A one-day window was maintained between each test to avoid intertest effects. This timeline and methodology are based on a published study [22].

### 4.8. Open-Field Test

The open-field test was used to assess locomotor activity and anxious behavior. The open field was 40 cm long × 40 cm wide × 30 cm high. Each mouse was allowed to explore the field for 6 min. Its activity was recorded and analyzed with Smart 3.0 software (Version 3.0.06) to determine the total distance traveled, the speed, and the number of entries to the center and peripheral zones. At the beginning of each test, each mouse was placed in the same corner of the arena.

### 4.9. Elevated Plus Maze

Each mouse was placed in the center of the elevated-plus maze, with its nose directed toward the same closed arm, and allowed to explore it for 6 min. The session was recorded and analyzed with Smart 3.0 software (Version 3.0.06) to determine the total distance traveled, the number of entries to each arm, and the distance traveled in each arm.

### 4.10. Marble Burying

The marble-burying test is used to detect anxiety-like behavior, which correlates with the number of marbles buried [23]. Corncob bedding material (5 cm deep) was placed inside a standard cage. Twenty marbles were evenly distributed across the bedding. Then the mouse was placed in the cage for 30 min. The number of unburied marbles (where less than half of the marble’s height was under the bedding) was determined.

### 4.11. Light/Dark Box

This test is based on a mouse’s natural conflict between seeking protection and exploring a novel environment [24]. The light/dark box consisted of two chambers: an open field and a dark box. Each mouse was placed in the dark box and allowed to explore the light and dark chambers for 10 min. The total distance traveled and the number of entries to the light box were determined.

### 4.12. Water Maze

The water maze consisted of a circular tank (120 cm in diameter) filled with water containing titanium dioxide so that it was opaque. A circular escape platform (5 cm in diameter) was placed 1 cm below the surface of the water. Each mouse was trained for 5 days. Each day, the mouse was released in each quadrant from a start position facing the tank wall. Each mouse was given 1 min to find the hidden platform. When it could not find the platform, it was led to it and allowed to sit on it to memorize the location. The escape latency was determined on day 6. The times spent in each quadrant and the zone containing the platform were determined.

### 4.13. Blood Pressure Monitoring

An automated CODA non-invasive blood pressure system with a tail cuff (Kent Scientific, Torrington, CT, USA) was used to measure blood pressure. The mouse was placed on a warming platform to maintain its body temperature. After the mouse was positioned in the holder, the appropriate-sized cuff was wrapped tightly over the tail. This blood pressure system displays 6 blood pressure measurements for each reading. 6 mice from each group were subjected to blood pressure readings, which were repeated three times.

### 4.14. ARN1468 Injection

The experiments involved 30 male *APP*/*PS1* mice that weighed 28–32 g. They were randomly assigned to three groups: *APP*/*PS1* mice fed a NS diet (the AD group), *APP*/*PS1* mice fed a HS diet (the AD+HS group), and *APP*/*PS1* mice fed a HS diet and injected with ARN1468 (the AD+HS+ARN1468 group). The mice were fed a HS diet for 4 months and then had a cannula implanted so that ARN1468 could be injected directly into the brain. Each mouse was anesthetized with tribromoethanol (100 mg/kg, 0.25%, intraperitoneal) and fixed in a stereotaxic apparatus. The skull was exposed, and a cannula was implanted in the CA1 region, specifically 1 mm caudal to bregma, 1.3 mm lateral to the midline, and 2.5 mm ventral to bregma. The system was fixed to the skull via stainless steel screws and dental cement. Then the skin was sutured, and the cannula was exposed. Each mouse was allowed to recover separately for 48 h in a metal cage with free access to water and food. For the infusion, the mouse was anesthetized with sevoflurane. ARN1468 (10 mg/kg) or saline in a total volume of 2 µL was injected slowly (0.25 µL/min) through a system consisting of a 30G needle inserted into the guide cannula and connected through a polyethylene catheter to a 5 μL Hamilton micro-syringe. Immediately after the injection, the mouse was placed on a warm pad for a few minutes for recovery from anesthesia and then put back in its home cage.

### 4.15. RNA-seq

The RNA purity and concentration were evaluated using a NanoDrop 2000 spectrophotometer (Thermo Scientific, Waltham, MA, USA). RNA integrity was assessed using the Agilent 2100 Bioanalyzer (Agilent Technologies, CA, USA). RNA libraries were constructed using a TruSeq Stranded mRNA LT Sample Prep Kit (Illumina, San Diego, CA, USA) according to the manufacturer’s instructions. The transcriptome sequencing and analysis were conducted by OE Biotech Co., Ltd. (Shanghai, China).

### 4.16. RNA Isolation and Quantitative Real-Time Polymerase Chain Reaction (qRT-PCR)

RNA was isolated from the brain with the TRIzol reagent (Roche, Basel, Switzerland) following the manufacturer’s protocol. Complementary DNA (cDNA) was synthesized using a Reverse Transcription System (Thermo Fisher Scientific, Waltham, MA, USA). qRT-PCR was conducted using primer sets for the indicated transcripts and the SYBR Green master mix (Thermo Fisher Scientific, Waltham, MA, USA).

### 4.17. Western Blotting

Protein was isolated from the hippocampus using a lysis buffer containing protease and phosphatase inhibitors (Beyotime, Shanghai, China). Proteins were separated on a 10% sodium dodecyl sulfate (SDS) polyacrylamide gel and then transferred to a 0.22 μM polyvinylidene fluoride membrane (Millipore, Burlington, MA, USA). The membrane was incubated with rabbit anti-caspase 3 (1:1000, #9662, CST, Danvers, MA, USA) [45], rabbit anti-Bax (1:1000, #2772, CST, MA, USA) [46], rabbit anti-Syn1 (7H10G6) (1:200, NBP2-61895,Novus, Chesterfield, CA,USA) [47], rabbit anti-β-tubulin3 (1:3000, #2128, CST) [48], mouse anti-β-actin (1:10000, A5316, Sigma, MI, USA), rabbit anti-GAPDH (14C10) (1: 1000, #2118, CST) [49],rabbit anti-serpina3N (1:500, #AF4709, R&D, Minneapolis, MN, USA) [50,51], and rabbit anti-LC3A/B (#4108, CST, MA, USA) overnight at 4 °C. After this incubation, the membrane was incubated with goat anti-mouse IgG conjugated to horseradish peroxidase (HRP) (1:10,000, 12-349, Sigma, Livonia, MI, USA) or goat anti-rabbit IgG conjugated to HRP (1:10,000, SAB2108825, Sigma, MI, USA) for 2 h at room temperature. The ECL chemiluminescence reagent (Beyotime, Shanghai, China) was used to visualize the proteins.

### 4.18. Docking Simulations

Serpina3n was retrieved from the PDB database. PyMOL version 3.1 software was utilized to perform dehydration, ligand removal, and other operations on the receptor protein. Auto Dock Tools was used to modify the receptor protein by adding hydrogens and balancing charges and to convert the receptor protein and small-molecule ligand to the pdbqt format. AutoDock Vina 1.1.2 was used to perform molecular docking between the receptor protein and the small-molecule ligand, and PLIP was used to analyze the docking results. Finally, PyMOL was used to visualize the docking results.

### 4.19. Statistics

GraphPad Prism 8.0 was used for statistical analysis. The data are presented as the mean ± standard error of the mean. After assessing the normality of the data, Student’s *t*-test was used for analysis, with *P* < 0.05 considered to indicate a statistically significant difference.

## 5. Conclusions

In summary, we demonstrated that a HS diet could induce neuronal loss, suppress the expression of synaptic proteins, lead to Aβ plaque aggregation in the hippocampus, and cause anxiety-like behavior in *APP*/*PS1* mice. The expression of most *serpin* family members increased in the HS group, among which serpina3n plays a key role. By inhibiting serpina3n, the anxiety-like behavior, neuronal loss in the hippocampus, and apoptosis were attenuated. This study indicates that HS consumption induces neuronal loss and anxiety-like behavior through the upregulation of serpina3n. Although the exact mechanisms are still not certain, our study provides novel insights into the role of the serpin family in the effect of HS intake on anxiety and neuronal loss in the hippocampus. Our study draws attention to the importance of reducing daily salt intake, especially for patients with Alzheimer’s disease.

## Data Availability

All the data are available on reasonable request from the corresponding author.
